# The Core Outcome DEvelopment for Carrier Screening (CODECS) study: protocol for development of a core outcome set

**DOI:** 10.1186/s13063-021-05439-7

**Published:** 2021-07-22

**Authors:** Ebony Richardson, Alison McEwen, Toby Newton-John, Karine Manera, Chris Jacobs

**Affiliations:** 1 Graduate School of Health, University of Technology Sydney, Building 20, 100 Broadway, Chippendale, Sydney, NSW 2008 Australia; 2 Sydney School of Public Health, The University of Sydney, Edward Ford Building, A27 Fisher Rd, Sydney, NSW 2006 Australia

**Keywords:** Core outcome set, Reproductive genetic carrier screening, Genetic counselling, Patient-reported outcomes, Qualitative research, Delphi survey, Outcome reporting

## Abstract

**Background:**

Reproductive genetic carrier screening is a type of genetic testing available to those planning a pregnancy, or during their first trimester, to understand their risk of having a child with a severe genetic condition. There is a lack of consensus for ‘what to measure’ in studies on this intervention, leading to heterogeneity in choice of outcomes and methods of measurement. Such outcome heterogeneity has implications for the quality and comparability of these studies and has led to a lack of robust research evidence in the literature to inform policy and decision-making around the offer of this screening. As reproductive genetic carrier screening becomes increasingly accessible within the general population, it is timely to investigate the outcomes of this intervention.

**Objectives:**

The development of a core outcome set is an established methodology to address issues with outcome heterogeneity in research. We aim to develop a core outcome set for reproductive genetic carrier screening to clarify and standardise outcomes for research and practice.

**Methods:**

In accordance with guidance from the COMET (Core Outcome Measures in Effectiveness Trials) Initiative, this study will consist of five steps: (i) a systematic review of quantitative studies, using narrative synthesis to identify previously reported outcomes, their definitions, and methods of measurement; (ii) a systematic review of qualitative studies using content analysis to identify excerpts related to patient experience and perspectives that can be interpreted as outcomes; (iii) semi-structured focus groups and interviews with patients who have undertaken reproductive genetic carrier screening to identify outcomes of importance to them; (iv) Delphi survey of key stakeholders, including patients, clinicians, and researchers, to refine and prioritise the list of outcomes generated from the previous steps; and (v) a virtual consensus meeting with a purposive sample of key stakeholders to finalise the core outcome set for reporting.

**Discussion:**

This protocol outlines the core outcome set development process and its novel application in the setting of genetic testing. This core outcome set will support the standardisation of outcome reporting in reproductive carrier screening research and contribute to an evolving literature on outcomes to evaluate genetic testing and genetic counselling as health interventions.

**COMET core outcome set registration:**

http://www.comet-initiative.org/Studies/Details/1381.

## Background

Genetic testing is an increasingly common health intervention across numerous clinical settings and is recognised to hold vast potential for improving patient care. Genetic counselling is the process that surrounds the consideration of genetic risk, encompassing many aspects directly related to genetic testing. Genetic counselling can be performed by genetic counsellors educated at the post-graduate level and regulated by a professional body. Genetic counselling provided by genetic counsellors includes a number of aspects separate to whether a genetic test is undertaken, for example, facilitating understanding, providing individual and family support, and assisting clients with adjustment to genetic risk [1]. However, aspects of genetic counselling are also undertaken by a range of other health professionals involved in patient care. As the processes of genetic testing and genetic counselling are intricately entwined, so too are the health-related outcomes that are measured in research in this area. Most research in the field of genetics does not distinguish between outcomes of genetic testing and genetic counselling; therefore, both will be considered and referred to herein.

One of the most widely adopted genetic tests worldwide is reproductive genetic carrier screening (RGCS), which provides individuals and couples with information about their risk of having a child with a genetic condition before or during early pregnancy [2]. RGCS identifies carriers of recessively inherited conditions (autosomal recessive or X-linked), such as cystic fibrosis, spinal muscular atrophy, and fragile X syndrome. These conditions often arise unexpectedly and carriers are, in most instances, asymptomatic. Since there is usually only an increased reproductive risk if a carrier chances to partner with a carrier of the same condition, most couples that have an affected child will not have an existing family history that could have forewarned of their risk. Recessively inherited conditions are individually rare but when combined are estimated to affect at least 30 in every 10,000 or 0.3% of births [3–5]. Based on this birth prevalence, it is estimated that 1–2% of couples will be at risk for having a child affected with a genetic condition, and this number can be much higher in consanguineous populations [6]. Of those at an increased risk, the likelihood of having an affected child ranges from 25 to 50% in each pregnancy, depending on the specific condition. The intent of RGCS is to provide couples who are at increased risk with information to allow them to make informed reproductive decisions. Those who are aware of their risk can choose to pursue prenatal diagnosis during pregnancy, opt for in vitro fertilisation (IVF) with preimplantation genetic diagnosis or the use of a donor gamete, consider adoption, or pursue pregnancy without any intervention and diagnose postnatally if desired. For this protocol, individuals and couples undertaking RGCS will be referred to as patients; however, we acknowledge that these will be largely healthy adults, most of which will not go on to require significant medical follow-up as a result of their carrier screening results.

Carrier screening programmes have been implemented since the 1970s in populations that have increased carrier frequencies for certain conditions, with targeted testing of only the conditions relevant to that population. Such conditions include but are not limited to Tay-Sachs disease in the Ashkenazi Jewish population, and thalassaemia and other inherited haemoglobinopathies across a range of ethnicities [7–9]. These programmes pre-dated our ability to identify carriers through genetic testing, instead relying on biochemical assays, with cystic fibrosis being one of the first conditions to have a screening programme based on molecular methods introduced in the 1990s [10]. Early carrier screening programmes typically focused on one genetic condition; however, recent advancements in genetic technologies have enabled a shift in the breadth of RGCS. Next-generation sequencing has facilitated the development of ‘expanded’ panels that analyse hundreds to thousands of genetic conditions in a single laboratory test. These expanded panels are broadly available to the general population, and whilst they have predominantly been commercial offerings to date, largely limiting their uptake to high-income groups, there are emerging efforts internationally to support equitable access to expanded screening [11]. There are now a range of ways in which individuals and couples may access RGCS, including community screening programmes in increased risk populations, attending public or private prenatal services during early pregnancy, or accessing preconception care through general practitioners or genetic counsellors in the public or private sectors.

There is increasing support for RGCS to be offered widely. In 2016, the Society of Obstetricians and Gynaecologists of Canada Genetics Committee and the Canadian College of Medical Geneticists Clinical Practice Committee (SOGC-CCMG) released a joint practice recommendation supporting the discussion of RGCS with all women/families considering pregnancy or at their first prenatal visit [12]. This advice was closely followed by a similar practice recommendation from the American College of Obstetricians and Gynaecologists (ACOG) in 2017 [13, 14]. These international organisations were amongst the first to support the widespread offer of RGCS outside of increased risk populations, with The Royal Australian and New Zealand College of Obstetricians and Gynaecologists (RANZCOG) following suit in March 2019 [15]. With this building momentum, it is a pivotal time to address research efforts to evaluate the impact of RGCS.

As with other areas of medicine, one of the aims of research in the field of genetics is to understand the benefits and harms of genetic testing as a health intervention. This is most often achieved by measuring the impact of a genetic test on patient outcomes when it is utilised in clinical practice; however, this is acknowledged to be challenging [16, 17]. There is an established literature aiming to demonstrate the effectiveness of clinical genetics services, genetic counselling, and genetic testing, and systematic reviews have overall demonstrated a modest positive impact [1, 18–23]. A problem that has arisen frequently in the genetics literature is comparability across studies, with heterogeneity in the choice of outcomes and method of measurement. Often, studies measure the same or similar concepts, such as psychological impact, but vary in the specific outcome that they report within this broad domain, utilise different measurement tools, and measure the outcome at variable time points. When outcome heterogeneity exists, the ability to directly compare and contrast the results of studies is hindered, and combining results, such as in a meta-analysis, becomes unreliable. This issue has been highlighted in research and commentary on the outcomes of genetic testing and genetic counselling and is becoming a focus of many discussions within the field [1, 18].

Another issue noted in genetics research is the propensity for observational study designs due to the challenges of including a comparison group in the clinical setting. Very few studies on RGCS are experimental in design, with only a handful of randomised controlled trials. Observational study designs are well-recognised to have a lower standard of methodological rigour, with a number of potential problems that may lead to biasing of results [24]. One such issue is that there is not a requirement or tendency to publish a protocol outlining the outcomes that will be measured. This introduces a risk of reporting bias as there is a lack of accountability for publishing all outcomes, regardless of whether they support the author’s position or reach statistical significance. There is also a great deal of variability in the inclusion of patient-reported outcomes, which are important for ensuring that the results of the research are relevant to patients. A small number of systematic reviews have been conducted in the field of RGCS, focusing on carrier screening for specific conditions. Those reviews that address data analysis and risk of bias in their methods identified issues with outcome heterogeneity, study design, and overall quality of evidence, whilst others that did not specifically address these issues performed narrative syntheses, which is indicative that a meta-analysis was not possible with the available data [23, 25–28].

We propose developing a core outcome set (COS) for RGCS. A COS is an agreed minimum set of outcomes that should be measured and reported in all studies on a particular topic [29]. The development of a COS applies a rigorous approach to defining outcomes that are relevant to all key stakeholders of a health intervention. This approach aims to minimise the heterogeneity in outcomes that are measured by different researchers, and as a result, maximise the ability to compare and combine studies in meta-analysis or other data synthesis approaches. Defining a COS also reduces the likelihood of reporting bias by ensuring that, at the very least, the core outcomes would be reported in all studies on an intervention. The incorporation of individuals who have had RGCS, clinicians involved in their care, and researchers and policy-makers guiding practice in this area in the development of this COS will ensure that outcomes are relevant to all stakeholders.

The Core Outcome DEvelopment in Carrier Screening (CODECS) study will apply the methodology outlined by the COMET (Core Outcomes Measures in Effectiveness Trials) Initiative to develop a COS for RGCS. To our knowledge, this study will be the first example of a COS aimed at standardising the reporting of outcomes in studies on a genetic testing intervention.

## Methods/design

### Scope

The methodology defined by the COMET (Core Outcomes Measures in Effectiveness Trials) Initiative and the Core Outcome Set-STAndardised Protocol Items (COS-STAP) Statement will inform this protocol [29, 30]. The COMET database was searched to confirm that there were no overlapping projects and the CODECS study subsequently registered (http://www.comet-initiative.org/Studies/Details/1381). The PICO framework is recommended by the COMET initiative for defining the scope of a core outcome development study, using the first three elements of population, intervention, and comparator [31]. The population that this COS is being developed for incorporates any individual or couple that is offered genetic carrier screening to inform their current or future reproductive decisions. This may be offered as population screening in increased risk populations as well as the general population and includes school, community, preconception, and prenatal programmes. This COS is not intended to cover cascade carrier screening in family members following the diagnosis of a genetic disease in a family member.

The definition of the intervention includes RGCS via targeted single-gene or small gene panels, through to pan-ethnic expanded carrier screening panels and virtual panels from whole-genome sequencing. The intervention encompasses pre-test genetic counselling, genetic testing, and post-test result management. Molecular genetic testing methodologies are the predominant laboratory method of carrier screening currently. However, some programmes do remain reliant on biochemical methods to triage access to molecular genetic panels. An example of this is haemoglobinopathy screening programmes, which use results of mean corpuscular volume (MCV) and mean corpuscular haemoglobin (MCH) ± anaemia, to triage which individuals will be screened using molecular methods. This COS is intended to be applicable to molecular and combined biochemical/molecular methods. A comparator is not expected to be appropriate for most RGCS programmes. However, where appropriate, we will include comparators such as control populations (no RGCS testing) and targeted versus expanded interventions (single-gene or small panels compared to expanded panels). This COS is intended to be applicable to all population-based RGCS scenarios and is being developed to take into account the significant variability in screening approaches used internationally.

This COS is being developed for use in research on RGCS, as well as in clinical practice. The majority of research in this area involves observational study designs assessing the impact of RGCS after it has already been implemented into clinical practice, and it is only recently that there has been a shift in the literature towards more rigorous study methodologies using randomised controlled designs. Therefore, it was decided that separating out the research and clinical contexts was not possible for this COS.

**Fig. 1:**
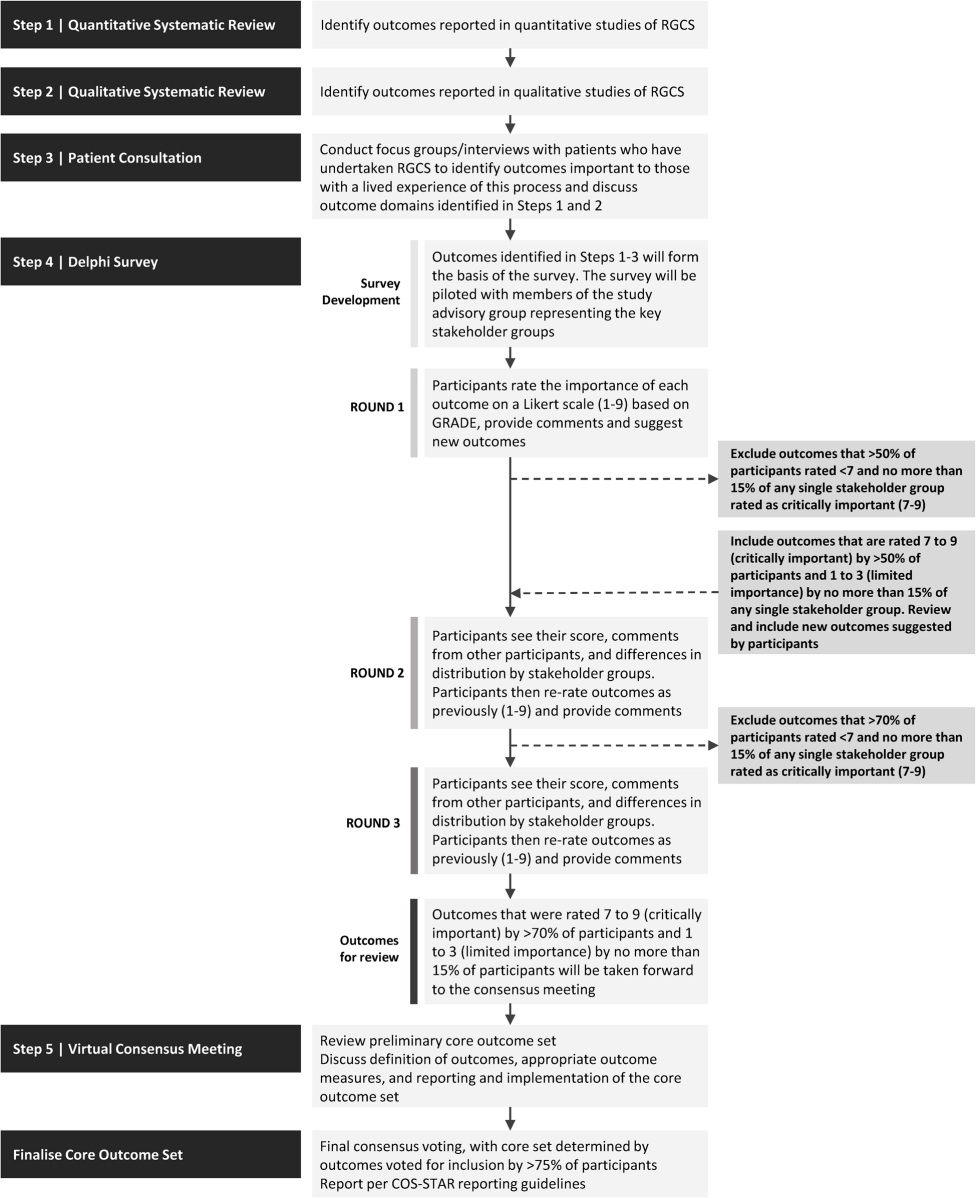
Study overview diagram

The CODECS study involves five steps: a systematic review of quantitative literature, a systematic review of qualitative literature, semi-structured focus groups/interviews with patient stakeholders, an international online Delphi survey, and a virtual consensus meeting (Fig. 1).

### Step I—Systematic review of outcomes reported in quantitative studies on reproductive genetic carrier screening

A systematic review of the literature will be conducted to identify outcomes and their method of measurement in studies evaluating an offer of RGCS. These will form the basis of the preliminary list of outcomes that will be reviewed and refined during the consensus process. The full protocol for this systematic review is published on the international prospective register of systematic reviews, PROSPERO (CRD42019140793).

#### Study selection

MEDLINE, CINAHL, PsycINFO, and EMBASE will be searched for quantitative, qualitative, and mixed methods studies. The quantitative and qualitative studies identified will be reviewed separately to account for the different approaches needed to extract the outcomes. Step I will include studies that are solely quantitative, or for mixed methods studies, the portion of quantitative data. A percentage of title and abstract screening will be performed independently by two reviewers until inter-rater reliability of >85% is achieved, after which the remainder will be screened by ER only due to resource limitations. The full-text screening will be similarly performed. Any disagreement on the eligibility of studies will be resolved through discussion with a third reviewer.

All peer-reviewed published studies where reproductive genetic carrier screening has been offered as a health intervention will be eligible for inclusion. Studies that are primarily evaluating laboratory test methodology, are not primary research, where the context of testing is not primarily related to RGCS (for example, newborn screening, cascade carrier screening), or that are not available in English, will be excluded.

#### Data extraction

Outcomes will be extracted from eligible studies from the last 5 years to form a preliminary list; the review will then proceed to the previous 5 years and compare outcomes to the preliminary list. If no additional outcomes are identified, the review will be considered complete after this 10-year period; however, further cycles will be conducted if additional outcomes continue to be identified. This methodology is per the guidance of the COMET handbook, suitable for situations where the size of the review would be unmanageable if conducted in full [29]. A guideline for data extraction has been developed and will be piloted with two independent reviewers for 20% of studies. Outcomes, and where supplied, their definition, method of measurement, and time point, will be extracted verbatim from studies using NVivo software. The primary outcome, if specified, will be noted. In addition, study type, target population, intervention type, screening approach, and other basic study characteristics will be extracted.

#### Quality assessment

The quality of the included studies will be scored using the QualSyst tool by the primary reviewer (ER) [32]. In the context of a systematic review of outcomes where the aim is to determine all published outcomes regardless of study quality, the assessment of bias will not be used as grounds for exclusion but rather to give an overall evaluation of the quality of studies within the RGCS literature.

#### Analysis and presentation of results

A narrative synthesis will be conducted on data extracted from quantitative studies, with outcomes to be grouped within domains and mapped to the COMET taxonomy [33, 34]. The domains will be reviewed and discussed by the CODECS study management group. The number of different outcomes (including the method of measurement and time points) and the number of studies that assessed each outcome will be evaluated. Subgroup analyses will be considered to identify outcomes that may be specific to targeted and expanded carrier screening approaches.

### Step II: Systematic review of outcomes reported in qualitative studies on reproductive genetic carrier screening

The inclusion of patient perspectives is considered a minimum standard of the COS development process in accordance with the Core Outcome Set-STAndards for Development (COS-STAD) [35]. The COMET handbook suggests that the preliminary list of outcomes generated by a systematic review of the quantitative literature may be supplemented with additional domains derived from a review of the qualitative literature if time and resources allow [29]. We will apply the methodology developed by Gorst et al. to extract outcomes of importance to patients from the qualitative literature on RGCS [36]. We will compare the extracted outcomes identified from the qualitative literature with those identified from the quantitative literature. These outcomes will be used to identify gaps in knowledge or representation of patient groups to guide focus groups/interviews in step 3.

#### Study selection

The initial steps of the systematic review will be conducted in the same manner as the quantitative studies described above, diverging at the point of data extraction. Eligible studies will be those that utilise qualitative methodology or mixed methods studies involving a qualitative component.

#### Data extraction

It is not anticipated that any existing studies will have conducted qualitative research specifically for the purpose of identifying outcomes. Therefore, our approach to data extraction will be deductive, taking excerpts verbatim from the qualitative literature and deducing the outcome that they represent. Excerpts will be any text relating to how patients felt or were impacted by their experience of undertaking RGCS, including quotations and author’s interpretation of themes. Each relevant excerpt will be extracted as a node using NVivo software by the primary reviewer (ER) and checked by a second reviewer (CJ) to ensure that all relevant text excerpts have been extracted. Both reviewers will then independently interpret outcomes from 20% of studies and check these for agreement. A coding guideline will be developed and used by the primary reviewer (ER) to interpret the remaining excerpts.

#### Quality assessment

The quality of the included studies will be scored as described above for quantitative studies.

#### Analysis and presentation of results

We will draw on content analysis to conduct a narrative synthesis of the data from eligible studies [34]. This method will allow us to convert qualitative findings into frequency counts, permitting comparison with the findings of the quantitative literature. Outcomes will be grouped within domains and mapped to the COMET taxonomy as above. Each text excerpt and its deduced outcome will be independently categorised into the taxonomy by two reviewers (ER and CJ). All categorisations will be discussed until 100% agreement is reached, with reference back to the original article for context as needed. Some outcomes are expected to be relevant to multiple domains within the taxonomy, and where this occurs, they will be categorised under two domains as recommended in the taxonomy guidance [33].

### Step III: Semi-structured patient focus groups/interviews

We will conduct primary qualitative research with patients who have undertaken RGCS to identify outcomes important to those with a lived experience of this process. We will give participants a choice of attending a focus group or one-on-one in-depth interview. Focus groups are a valuable way to encourage participant interaction and enrich the sharing of their experiences; however, there are a number of factors that may influence the appropriateness of conducting focus groups [37]. It is possible that recruitment may be limited by factors related to the sensitivity of the research topic, in particular amongst participants that fall into the increased risk group and may feel uncomfortable relaying their experience in a group setting. Therefore, the option of one-on-one interviews will also be available and decided upon once recruitment is underway.

#### Participants and recruitment

We will recruit individuals or couples who have had RGCS in order to inform their reproductive decisions. Participant groups will be defined by two characteristics: their level of risk prior to RGCS (a priori) and their level of risk following results (a posteriori). A priori risk will be either average or increased. Average a priori risk will be defined as *the participant having no existing health concerns or family history to indicate an increased risk of being a carrier*. Increased a priori risk will be defined as *the participant having an existing factor such as ethnicity with a known increased frequency of carriers or a known family of a genetic condition that is included in the screening*. There are a number of potential outcomes of RGCS; however, a posteriori risk will be grouped into either low or increased reproductive risk. Low reproductive risk results are defined as those where neither member of a reproductive couple are found to be carriers of the same genetic condition, or where one member of a reproductive couple is found to be a carrier of an autosomal recessively inherited genetic condition but their reproductive partner is not a carrier of the same condition. Increased reproductive risk is defined as those where both members of a reproductive couple are found to be carriers of the same autosomal recessively inherited genetic conditions, or where the female reproductive partner is found to be a carrier of an X-linked genetic condition. We will aim to recruit 30 participants, with equal representation from each group.

To recruit an international and diverse sample with a range of experiences, we will circulate an expression of interest to participate in the research through a number of social media channels and parenting forums. Respondents who follow the link will be directed to an online survey on the Qualtrics platform to receive more information about the research and fill out basic demographic and screening questions to confirm their eligibility [38]. Eligible participants will be from countries that score > 50 on the corruption perceptions index, indicating that they are not vulnerable populations [39].

#### Data collection

Focus groups/interviews will be conducted using Zoom, an online audio- and video-conferencing platform [40] Recent research has indicated the viability of Zoom as a tool for the collection of qualitative data due to its ease of use, cost-effectiveness, data management, and security features [41]. Focus groups will be approximately 90 min, depending on the number of participants. One-on-one interviews will be up to 60 min in duration. An online platform has been chosen to facilitate international participation and reduce the inconvenience of travelling for participants. Focus groups/interviews will be audio- and video-recorded and transcribed verbatim. The focus group/interview schedule will begin with open questions to elicit patient experiences, after which more specific questions related to outcomes will be informed from the list of outcome domains generated from the systematic review steps above. Our data collection will draw on grounded theory, with data collection and analysis occurring concurrently and utilising constant comparison to refine data collection as the study progresses [42].

#### Data analysis

Using thematic analysis, the transcripts will be reviewed line by line and inductively coded to identify concepts [43]. Similar concepts will be grouped into themes and corresponding subthemes. These concepts/themes will reflect the perspectives, beliefs, and values of participants in regard to outcomes of reproductive carrier screening. To ensure that the complete range and depth of the data are included, at least two investigators will be involved in coding the data. Data collection will continue until data saturation is reached (the point at which no new themes are identified).

### Step IV: Delphi consensus survey

We will follow published principles of applying the Delphi process in the context of COS development [44]. This process will utilise a sequential, two- to three-round online survey with an internationally representative sampling of key stakeholders in the field.

#### Developing the survey

The preliminary list of outcomes generated from the previous steps will be reviewed by the research team to form the basis of the Delphi survey. Lay language summaries will be developed and presented together with the medical definitions to facilitate the participation of patients in this step of the COS development process. The Delphi survey will be generated using Qualtrics software and piloted with the study advisory group. Feedback will be incorporated into the survey structure, definitions and lay language summaries, and overall usability of the survey.

#### Participants and recruitment

Five key stakeholder groups with current or recent personal, clinical, research, or policy experience of RGCS will be targeted for the Delphi survey: patients (including both carriers and non-carriers identified through targeted or expanded screening), genetic health professionals (genetic counsellors and clinical geneticists), non-genetic health professionals (obstetrician/gynaecologists, midwives, general practitioners), and policy-makers.

No recommendations currently exist regarding sample size for a Delphi survey, with wide variability in the number of participants across Delphi surveys for COS development [45]. Decisions regarding sample size are based on the area of practice and feasibility of recruiting sufficient representation from each stakeholder group. As this is the first COS that we are aware of in the setting of genetic testing, we do not have a guide for how many stakeholders may be willing to participate in this process. We have adopted the approach of COS developers in the obstetric setting [46]. Equal representation of patient and professional perspectives is desirable; as such, we will aim to recruit at a minimum 50 patient participants and 50 participants from other professional stakeholder groups to the first round of the Delphi survey. In recognising natural rates of attrition in subsequent rounds, this number should allow sufficient representation through the three rounds of the Delphi process.

Our recruitment strategy incorporates diverse methods of identifying and recruiting participants to account for the range of key stakeholders that we are aiming to include. Expressions of interest to participate will be distributed through various channels: (1) patient participants from focus groups/interviews will be invited to participate in the Delphi process. We will also recruit through social media to reach our goal of 50 total patient participants; (2) researchers in the field will be purposively sampled based on first and last authors of papers included in our systematic reviews; (3) genetic and non-genetic health professionals will be purposively sampled based on professional networks of the research team and member lists of relevant professional organisations; (4) policy-makers will be purposively sampled from listed committee members on major practice recommendations related to RGCS. All participants will be encouraged to snowball information about the study to their networks to ensure a broad range of participants and experiences are captured. Participants who respond to expressions of interest through any of the above channels will be directed to an online survey on the Qualtrics platform to receive more information about the research and fill out basic demographic and screening questions to confirm their eligibility. Once eligibility is confirmed, they will receive the link to the Delphi survey, where they will be required to electronically indicate their consent before proceeding to the consensus questions. During the Delphi survey, recruitment may be targeted to groups that are under-represented to ensure balanced representation.

#### Data collection

In round 1, participants will be asked to rate each outcome on a 9-point Likert scale based on the degree of importance as recommended by the Grading of Recommendations Assessment, Development, and Evaluation (GRADE) working group [47]. Rating 1 to 3 will be interpreted as ‘limited importance’, 4 to 6 as ‘important, but not critical’, and 7 to 9 as ‘critical importance’. An option of ‘unsure’ will also be available. The sequence of questions will be randomised to minimise ordering bias. For each outcome, a free text box will be available for participants to provide feedback or comments. New outcomes can be suggested by participants at the end of round 1 and will be reviewed by the research team to determine if they are unique and not overlapping, wholly or partially, with an existing outcome. Those that are deemed to be suitable will be carried over to round 2.

There is a lack of agreement on the definition of consensus to be used when deciding which outcomes to include in the second round of a Delphi survey, and a wide range of thresholds have been utilised in COS development. Per the guidance in the COMET handbook, using less stringent criteria in earlier rounds and considering responses from individual stakeholder groups minimise the likelihood that outcomes that may have been rated higher in subsequent rounds after receiving feedback are not dropped too soon in the Delphi process [29]. We will adopt the definition utilised in a recent COS developed for surgery in oesophageal cancer, whereby criteria for inclusion in round 2 will be any outcomes that are rated 7 to 9 (critically important) by >50% of participants and 1 to 3 (limited importance) by no more than 15% of any single stakeholder group [48]. Results will be presented graphically to participants at the time of the second round of the survey along with their rating of each outcome and any representative comments provided by participants that indicate their reasoning. This will allow participants to compare their ratings to other participants and consider whether they would change their rating in the next round. Participants will then be directed to re-rate the outcomes that have been carried over from the first round, with a free text box once again available for them to explain their rating or respond to comments from other participants from round 1. More stringent criteria for consensus will be applied to determine if there is a need for a third round of the Delphi survey, with outcomes that are rated 7 to 9 (critically important) by >70% of participants and 1 to 3 (limited importance) by no more than 15% of any single stakeholder group.

A third round of the Delphi survey will be conducted if the number of outcomes remaining after the second round is too high to reasonably discuss at a consensus meeting. Criteria for inclusion may need to be adjusted at the time of the Delphi if sufficient reduction in outcomes is not being achieved, changes to which will be reported alongside the results of the Delphi survey. Following the final round of the Delphi survey (whether that is the second or third), outcomes that were rated 7 to 9 (critically important) by >70% of participants and 1 to 3 (limited importance) by no more than 15% of participants will be taken forward to the consensus meeting for consideration of inclusion in the final COS.

Each round of the survey will be open for a minimum of 4 weeks to provide participants with sufficient time to complete it. A maximum of 3 reminders will be sent to participants when 2 weeks, 1 week, and 1 day are remaining to complete the survey. At the end of round 2 of the Delphi survey, participants will be asked to indicate if they are interested in participating in the final consensus meeting.

#### Data analysis

We will summarise the overall distribution in ratings for outcomes across the rounds of the Delphi survey and the points at which outcomes were excluded from consideration. The mean and median will be calculated for each outcome. Data will be analysed in sub-groups to allow comparison between prioritisation of outcomes between health consumer participants and other stakeholder participants, and also between different subsets of the other stakeholder groups (for example, genetic health professionals versus non-genetic health professionals).

### Step V: Consensus meeting

We will host a virtual consensus meeting using Zoom. The purpose of this meeting will be to review the findings from steps 1–4 and discuss the outcomes for inclusion in an agreed-upon COS. Methods of measurement, implementation, and directions for further research will also be discussed if time allows.

#### Participants and recruitment

From the participants that complete the Delphi survey, a maximum of 15 (2–3 from each participant group) will be purposively selected from those that have indicated an interest in participating in the final virtual consensus meeting, ensuring equal representation across stakeholder groups. We may consider contacting Delphi participants that did not express interest at the conclusion of the Delphi, where there is insufficient representation from specific groups. Purposively selected participants will be sent an email per the contact details they have provided at an earlier stage to invite them to participate. Verbal consent will be obtained at the beginning of the session. The number of participants selected to participate in the final virtual consensus meeting is a pragmatic decision based on balancing sufficient representation to incorporate all perspectives with a manageable number of participants that gives everyone a chance to contribute.

#### Data collection and analysis

The conduct of the virtual consensus meeting will be dependent upon the number of outcomes that need to be discussed based on the results of the Delphi survey. Allowances will be made for multiple meetings to facilitate international participation across time zones, and where the number of outcomes to discuss is likely to exceed 1–2 h of discussion, the meeting may be split into two sessions. The virtual consensus meeting will consist of a voting system as well as open sections of discussion. An overview of the CODECS project results to date will be presented at the start of the meeting, followed by a proposal of each outcome that satisfied the inclusion criteria set out in the Delphi survey. Participants will have the opportunity to discuss each outcome before lodging a vote for its inclusion or exclusion, with outcomes that achieve >75% consensus being included. The core outcome set literature varies in its approach to consensus thresholds for the consensus meeting, with the majority setting a minimum of 70%. A slightly higher threshold of 75% is favoured by some authors as it allows for increased stringency in the final step of the consensus process and we have elected this approach [49, 50]. Results will be presented after the voting is complete, outlining which outcomes reach consensus for inclusion in the core outcome set. Those that reach consensus for exclusion or that have no consensus will be reviewed, with panel members having an opportunity to provide an opinion if they see a fundamental reason why they disagree with the exclusion of these outcomes. Should the number of outcomes that reach consensus for inclusion be unwieldy, we may consider a tiered approach for reporting of the core outcome set, as has been done by previous COS developers [51]. Should time allow, we will finish the meeting with a discussion focusing on the definition of outcomes, appropriate outcome measures, and reporting and implementation of the COS; however, these will need to be addressed in more detail in subsequent research. The discussion sections of the meeting will be transcribed verbatim and analysed using thematic analysis as described for the focus groups/interviews above.

#### Dissemination and implementation

This COS will be reported according to the COS-STAR reporting guidelines [52]. Efforts for dissemination and implementation will include publishing the COS in an open-access journal, presenting the findings at conferences of relevant professional organisations, sharing with clinical trial registries, and encouraging stakeholder participants to circulate the final COS to their professional networks internationally.

## Discussion

RGCS is one of the most widely available genetic tests internationally and has the potential to provide families with information about their reproductive risks and allow them to make informed decisions in family planning and pregnancy. As with many other types of genetic testing, it is not clear what outcomes are best to assess when considering the impact of RGCS, which has led to marked heterogeneity within the literature and hindered policy-makers in their attempts to utilise high-quality research evidence to support its implementation into routine clinical practice. Contingencies exist from a policy perspective in such cases, allowing expert consensus to be used to make practice recommendations; however, this does not address the underlying issues.

This study will provide researchers with guidance on which outcomes to include, at a minimum, in any study evaluating RGCS. As has been seen in other contexts, the application of a COS ensures that a minimum set of outcomes are routinely reported in all studies on a particular topic, allowing reliable comparisons across studies to be achieved. It also facilitates the combining of data where appropriate for use in meta-analyses to quantify outcomes. As the context of RGCS can be diverse, from single-gene panels through to expanded panels of hundreds to thousands of conditions, measuring a core set of outcomes across different contexts will allow direct comparison and have the potential to highlight differences that arise when targeted versus expanded screening is offered. Such comparisons may reveal benefits, risks, or challenges that may be specific to different contexts and allow for tailored approaches to implementation that address the individual needs of targeted versus expanded offers. Reporting bias is minimised by requiring that the COS is always reported as a minimum, meaning that even non-significant changes will be represented in the literature. Differences that do not reach significance in studies with small sample sizes may reach significance when combined in a meta-analysis. The COS will ensure that outcomes that are relevant to patients are incorporated in future studies. The development of this COS will have implications beyond RGCS, to other forms of genetic testing, and assist in ongoing efforts to define outcomes of genetic services and genetic counselling.

## Trial status

Protocol version 1.0, October 2020.

The development of this core outcome set is ongoing, with systematic reviews of the quantitative and qualitative literature complete. 

## Data Availability

Not applicable.

## Abbreviations

CODECS: Core Outcome DEvelopment for Carrier Screening
COMET: Core Outcome Measures in Effectiveness Trials
COS: Core outcome set(s)
RGCS: Reproductive Genetic Carrier Screening
